# Mobile Integrated Health Interventions for Older Adults: A Systematic Review

**DOI:** 10.1093/geroni/igad017

**Published:** 2023-03-01

**Authors:** Nathan Louras, Meghan Reading Turchioe, Leah Shafran Topaz, Michelle R Demetres, Melani Ellison, Jamie Abudu-Solo, Erik Blutinger, Kevin G Munjal, Brock Daniels, Ruth M Masterson Creber

**Affiliations:** Department of Emergency Medicine, University of Michigan, Ann Arbor, Michigan, USA; School of Nursing, Columbia University, New York, New York, USA; Department of Population Health Sciences, Weill Cornell Medicine, New York, New York, USA; Samuel J. Wood Library and C.V. Starr Biomedical Information Center, Weill Cornell Medical College, New York, New York, USA; Department of Population Health Sciences, Weill Cornell Medicine, New York, New York, USA; Department of Population Health Sciences, Weill Cornell Medicine, New York, New York, USA; Department of Emergency Medicine, Mount Sinai Icahn School of Medicine, New York, New York, USA; Department of Emergency Medicine, Mount Sinai Icahn School of Medicine, New York, New York, USA; Department of Emergency Medicine, Weill Cornell Medicine, New York, New York, USA; School of Nursing, Columbia University, New York, New York, USA

**Keywords:** Health care systems and management (telehealth), Home, and community, based care and services, Information technology

## Abstract

**Background and Objectives:**

Mobile integrated health (MIH) interventions have not been well described in older adult populations. The objective of this systematic review was to evaluate the characteristics and effectiveness of MIH programs on health-related outcomes among older adults.

**Research Design and Methods:**

We searched Ovid MEDLINE, Ovid EMBASE, CINAHL, AgeLine, Social Work Abstracts, and The Cochrane Library through June 2021 for randomized controlled trials or cohort studies evaluating MIH among adults aged 65 and older in the general community. Studies were screened for eligibility against predefined inclusion/exclusion criteria. Using at least 2 independent reviewers, quality was appraised using the Downs and Black checklist and study characteristics and findings were synthesized and evaluated for potential bias.

**Results:**

Screening of 2,160 records identified 15 studies. The mean age of participants was 67 years. The MIH interventions varied in their focus, community paramedic training, types of assessments and interventions delivered, physician oversight, use of telemedicine, and post-visit follow-up. Studies reported significant reductions in emergency call volume (5 studies) and immediate emergency department (ED) transports (3 studies). The 3 studies examining subsequent ED visits and 4 studies examining readmission rates reported mixed results. Studies reported low adverse event rates (5 studies), high patient and provider satisfaction (5 studies), and costs equivalent to or less than usual paramedic care (3 studies).

**Discussion and Implications:**

There is wide variability in MIH provider training, program coordination, and quality-based metrics, creating heterogeneity that make definitive conclusions challenging. Nonetheless, studies suggest MIH reduces emergency call volume and ED transport rates while improving patient experience and reducing overall health care costs.

**Translational significance:** This review investigates the potential for a model of prehospital care, mobile integrated health (MIH), to improve outcomes for older adults. MIH programs allow community paramedics and other health professionals to assess, diagnose, and treat individuals in their homes within defined scopes and care protocols. Thus, older adults can receive urgent medical care through MIH without being transported to the emergency department and admitted to the hospital. In this review, we found several promising benefits of these programs for older adults.

Older adults often have complex medical issues and psychosocial vulnerabilities, placing them at increased risk for adverse outcomes secondary to hospitalization including functional decline, pressure injuries, falls, and other factors (Evans et al., 2014; Mudge et al., 2019). At the same time, health care systems are facing mounting pressure to reduce preventable emergency department (ED) visits and unplanned hospitalizations for older adults. Adults over the age of 75 are estimated to have 60 visits per 100 persons compared to all other age groups over 1-year-old (Cairns, 2021). The persistent generalized risk for adverse health outcomes following a hospitalization, known as posthospital syndrome (Krumholz, 2013) as well as fragmentation of follow-up care (DeVore et al., 2016) and home services postdischarge (Demiralp et al., 2021) create cycles of rehospitalization that can be difficult to disrupt.

Mobile integrated health (MIH) is a rapidly evolving care-delivery model using patient-centered, mobile resources in the out-of-hospital environment. It may include, but is not limited to, services such as providing telephone advice to 9-1-1 callers instead of resource dispatch; providing community paramedicine care, chronic disease management, preventive care, or postdischarge follow-up visits; or transport or referral to a broad spectrum of appropriate care, not limited to hospital EDs (Perina et al., 2016). The goal of MIH is to deliver high-quality and cost-effective out-of-hospital care in an effort to reduce unnecessary ED visits and unplanned hospitalizations (Xie et al., 2021). MIH programs are able to send licensed health care professionals, such as a traditional emergency medical service (EMS) agency, community paramedics (CPs), or advanced practitioners to the homes of medically complex patients at high-risk for readmission to provide remote care. Some programs utilize an on-demand telemedicine component to allow for direct visual evaluation or consultations with physicians (Figure 1; HealthIT.gov, 2020). Critically ill patients can be immediately identified and transferred to the ED, while nonemergent medical needs are treated in the home. CPs are trained to perform assessments and point-of-care testing, administer medications, assess home safety, and provide patient education among other tasks (Chellappa et al., 2018; Choi et al., 2016; Pang et al., 2019).

**Figure 1.**
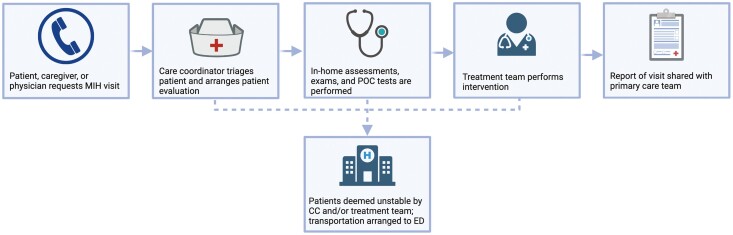
Common workflow of mobile integrated health (MIH) interventions. CC = care coordinator; ED = emergency department; POC = point-of-care.

MIH has been recognized, internationally, as a strategy to safely reduce avoidable ED visits, optimize quality of life, and extend the time older adults are able to live independently at home (Dainty et al., 2018). To date, hundreds of MIH programs have been established in the United States (Zavadsky, 2015), and yet little is known about the impact of MIH on older adults across a number of domains (Elbaz et al., 2021; Lam et al., 2020).

Prior systematic reviews of studies evaluating MIH programs have focused mainly on general adult populations (Chan et al., 2019) or older adults living in long-term care facilities (van Vuuren et al., 2021). A summary of studies evaluating MIH, specifically in the older adult population, is needed to understand the potential value these programs may have in improving outcomes for this group of adults with unique needs. Thus, in this systematic review our objective is to describe the evidence on the effectiveness of MIH programs on multiple health-related outcomes among adults aged 65 and older.

## Method

This review adhered to the Preferred Reporting Items for Systematic Reviews and Meta-Analyses (PRISMA 2020) guidelines (Page et al., 2021).

### Search Strategy

A medical librarian performed comprehensive searches to identify studies that addressed community paramedicine/MIH interventions among older adult populations. Searches were run on December 7, 2021, in the following databases: Ovid MEDLINE (ALL—1946 to Present); Ovid EMBASE (1974 to present); CINAHL (EBSCO); and The Cochrane Library (Wiley); AgeLine (EBSCO); Social Work Abstracts (Ovid). The search strategy included all appropriate controlled vocabulary and keywords for the concept of “community paramedicine.” The full search strategies for all databases are available in Supplementary Material. To limit publication bias, there were no language, publication date, or article type restrictions on the search strategy.

### Study Selection

Retrieved studies were screened for inclusion using Covidence systematic review software. Titles and abstracts were reviewed against predefined inclusion/exclusion criteria by two independent reviewers. Discrepancies were resolved by consensus. For final inclusion, full text was then retrieved and screened by two independent reviewers. Articles considered for inclusion were: (1) described a community paramedicine/MIH intervention, (2) focused on an older adult population and/or mean age of participants was 65 years or older, and (3) reported health and health care outcomes of the intervention. Excluded studies were: (1) duplicate articles, (2) abstracts only or non-peer-reviewed source, (3) published before 2015, (4) not available in English, (5) not reporting on original research (i.e., perspective article), (6) not reporting on an MIH intervention, and (7) not focused on an older adult population and/or mean age <65.

### Data Extraction

Data extraction was performed independently in duplicate with predefined, standardized templates. Data points defined for extraction were: year; study location and urbanicity; study design; intervention description; comparator arm (if used); sample description, including demographic characteristics of age, sex, race/ethnicity, and income, when reported; outcome measures; and study results. Disagreements were resolved through discussion.

### Data Synthesis

All included studies were considered eligible for synthesis. Data from the extraction form were tabulated. The synthesis occurred through discussion among team members. The synthesis included condensing similar information from the data extraction form into concise tables, and describing trends noted among the studies. No quantitative synthesis of results took place. Notes on the discussion regarding trends were transformed into the narrative description of the results by the first authors.

### Methodological Quality

Quality appraisal was performed using the Downs and Black checklist, a tool to appraise the quality of both randomized and nonrandomized study designs (Downs & Black, 1998). Using the checklist, quality appraisal was performed by five independent reviewers, followed by a three-person panel analysis of all final papers to discuss checklist criteria and address all disagreements.

## Results

Study characteristics and major findings are described in Table 1 and Supplementary Tables 1–3.

**Table 1.** Study Characteristics and Quality Appraisal Results

Study	Location and urbanicity	Study design	MIH intervention and comparator (if applicable)	Participants	Primary outcome measures	Primary results	Evidence grade^a^
Abrashkin et al. (2019)	US (New York); urban and suburban	Prospective, single-arm observational study	Home-based CP intervention including a telephone or telemedicine consult with a physician	1,159 home-bound individuals with 2+ chronic conditions	ED transport rates overall and by acuity level	17.9% of all CP responses and 21.0% of high acuity responses resulted in transport to the ED	12
Abrashkin et al. (2021)	US (New York); suburban	Retrospective, two-arm observational study	Home-based CP intervention; comparing visits with a telephone-based physician consult vs a video-based physician consult	1,068 home-bound individuals with 2+ chronic conditions	ED transport rates by telephone and video visits	Video availability was not associated with a significant difference in the odds of ED transport (OR: 0.80; 95% CI: 0.62–1.03)	18
Agarwal et al. (2017)	Canada (Ontario); urban	Prospective, single-arm interventional study	CP clinic in senior housing building lobby providing risk assessments and routine wellness services	79 residents of low-income senior housing	EMS call volume Blood pressure Canadian Diabetes Risk (CANRISK) score	EMS call volume decreased 25% Systolic blood pressure decreased significantly by the participant’s third visit and diastolic by the fifth visit (*p* < .05) 15% of participants dropped one CANRISK category (e.g., decreased risk)	18
Agarwal et al. (2018)	Canada (Ontario); urban	Cluster RCT	Senior housing buildings with CP clinics in lobby providing risk assessments and routine wellness services vs buildings without CP clinics	1,092 residents of low-income senior housing living in six buildings (three randomized to CP)	Number of EMS calls per 100 apartment units per month	Adjusting for baseline calls and building pairs, mean monthly ambulance calls was significantly lower in the intervention buildings than in the control buildings (3.11 [*SD* 1.30] vs 3.99 [*SD* 1.17] calls per 100 units/month; difference: –0.88 calls, 95% CI: –0.45 to –1.30)	23
Agarwal et al. (2019)	Canada (Ontario); urban	Cluster RCT	Senior housing buildings with CP clinics in lobby providing risk assessments and routine wellness services vs buildings without CP clinics	4,081 residents of low-income senior housing living in 30 buildings (15 randomized to CP)	Number of EMS calls per 100 apartment units per month	ITT analysis: no difference in EMS calls (–0.37 calls [95% CI: –1.00 to 0.27] per 100 apt units/month) Sensitivity analysis excluding one outlier patient and buildings with significant setting changes: significantly fewer EMS calls among intervention vs control (–0.90 calls [95% CI: –1.54 to –0.26] per 100 apt units/month)	24
Brokmann et al. (2016)	Germany; urban and rural	Prospective, two-arm interventional study	Home-based telemedicine consult for EMS providers treating ACS vs historical matched controls	39 patients diagnosed with ACS and treated by tele-EMS	Quality of prehospital care of ACS compared to national guidelines	No significant difference in the correct handling of 12-lead ECG or administration of aspirin, heparin, or morphine. The correct handling of oxygen was significantly high in the intervention group (*n* = 29 vs *n* = 18; *p* = .007).	16
Feldman et al. (2021)	US (Pennsylvania); rural	Prospective, single-arm interventional study	Home-based prescheduled and urgent visits for 30 days after hospital discharge	40 patients with heart failure (stage C *n* = 20; stage D *n* = 20)	All-cause 30-day readmissions	The incidence of 30-day all-cause readmissions was 15% for heart failure stage C patients, and 40% for heart failure stage D patients	16
Felzen et al. (2019)	Germany; urban	Retrospective, single-arm observational study	Home-based telemedicine consult for EMS providers treating ACS	6,265 patients diagnosed with ACS and treated by tele-EMS	Utilization (number of teleconsultations) Safety (adverse events) Technical performance of the system	The number of teleconsultations increased by 25.9 per quarter (95% CI: 9.1–42.6; *p* = .009) Six patients (0.10%; 95% CI: 0.04%–0.21%) experienced adverse events There were a small number of malfunctions with voice communications (0.3%, 95% CI: 0.2%–0.5%), data transmission (1.9%, 95% CI: 1.6%–2.3%), and complete system failures (0.3%, 95% CI: 0.2%–0.6%)	13
Jacobsohn et al. (2022)	US (New York and Wisconsin); suburban	Single-blind RCT	Prescheduled, self-management focused home visits by CP following hospitalization vs usual care	1,756 recently hospitalized community-dwelling older adults	ED visits within 30 days	MIH did not significantly affect odds of 30-day ED revisits (adjusted OR: 0.97, 95% CI: 0.72–1.30)	24
Kant et al. (2018)	US (Colorado); urbanicity not specified	Retrospective, observational case series	Home-based service using CPs, nurse practitioners, and physician assistants providing urgent care	35 senior clinic patients	Immediate transfer to ED ED visits and hospitalizations	Two patients (6%) were immediately transferred to ED Six patients (17%) had ED visits and five (14%) were hospitalized within 2 weeks of CP visit	12
Myers et al. (2020)	US (Wisconsin); rural	Retrospective, observational case series	Prescheduled home-based CP visits after referral from primary care doctors	32 patients identified as medically complex or high utilizers	ED visits Primary care clinic visits Hospitalizations	Statistically significant decrease in ED visits (–59.3%, *p* = .007) and primary care clinic visits (–53.3%, *p* = .006) compared to the 6 months preceding enrollment No difference in hospitalizations (*p* = .13)	18
Quatman-Yates (2021)	US (Ohio); suburban	Retrospective, single-arm interrupted time-series analysis	CP program designed to reduce fall-related EMS calls and transports through prevention activities	Patients who made 892 fall-related emergency calls (sample size not provided)	Fall-related calls Transports to the hospital	Significant reduction of fall-related calls (relative risk: 0.63 [95% CI: 0.4–0.75]) and fall-related transports (relative risk: 0.49 [95% CI: 0.27–0.64])	13
Roeper et al. (2018)	US (Florida); primarily urban	Retrospective, observational, case–control study	Prescheduled and urgent home-based MIH visits vs propensity score-matched control group	2,315 patients in a Florida-wide PPO who were (1) transitioning home after hospital, (2) high risk/chronically ill, or (3) needed palliative support	Cost savings	Over 6 months the net savings for MIH compared to usual care was $2,407,612	18
Shah et al. (2018)	US (New York and Wisconsin); suburban	Single-blind RCT	Prescheduled, self-management focused home visits by CP following hospitalization vs usual care	853 recently hospitalized community-dwelling older adults	Feasibility (visit and follow-up phone call completion) Acceptability (patient satisfaction) Fidelity to treatment protocol	Coaches successfully completed 84% of home visits and 86%–93% of three post-visit follow-up calls High acceptability endorsed by 76% of patients and 83% of caregivers Fidelity to the treatment protocol occurred in 88% of visits	17
Snooks et al. (2017)	UK; urban	Cluster RCT	Home-based, urgent CP care for older adults who have fallen vs usual paramedic care	4,655 older adults with a home-based fall and no concerning other symptoms reported during initial EMS call (i.e., chest pain)	Subsequent emergency events (composite outcome: EMS calls, ED visits, hospitalizations, death)	No difference when evaluated as composite outcome Significantly greater decrease in EMS calls in intervention vs control (adjusted difference: –0.0045; 95% CI: –0.0073% to –0.0017%)	25

*Notes*: ACS = acute coronary syndrome; CI = confidence interval; CP = community paramedic; ECG = electrocardiogram; ED = emergency department; EMS = emergency medical services; ITT = intention-to-treat; MIH = mobile integrated health; OR = odds ratio; PPO = Preferred Provider Organization; RCT = randomized controlled trial; UK = United Kingdom; US = United States.

^a^Evidence grade determined using the Downs and Black quality appraisal tool.

### Article Screening and Included Studies

**Figure 2.**
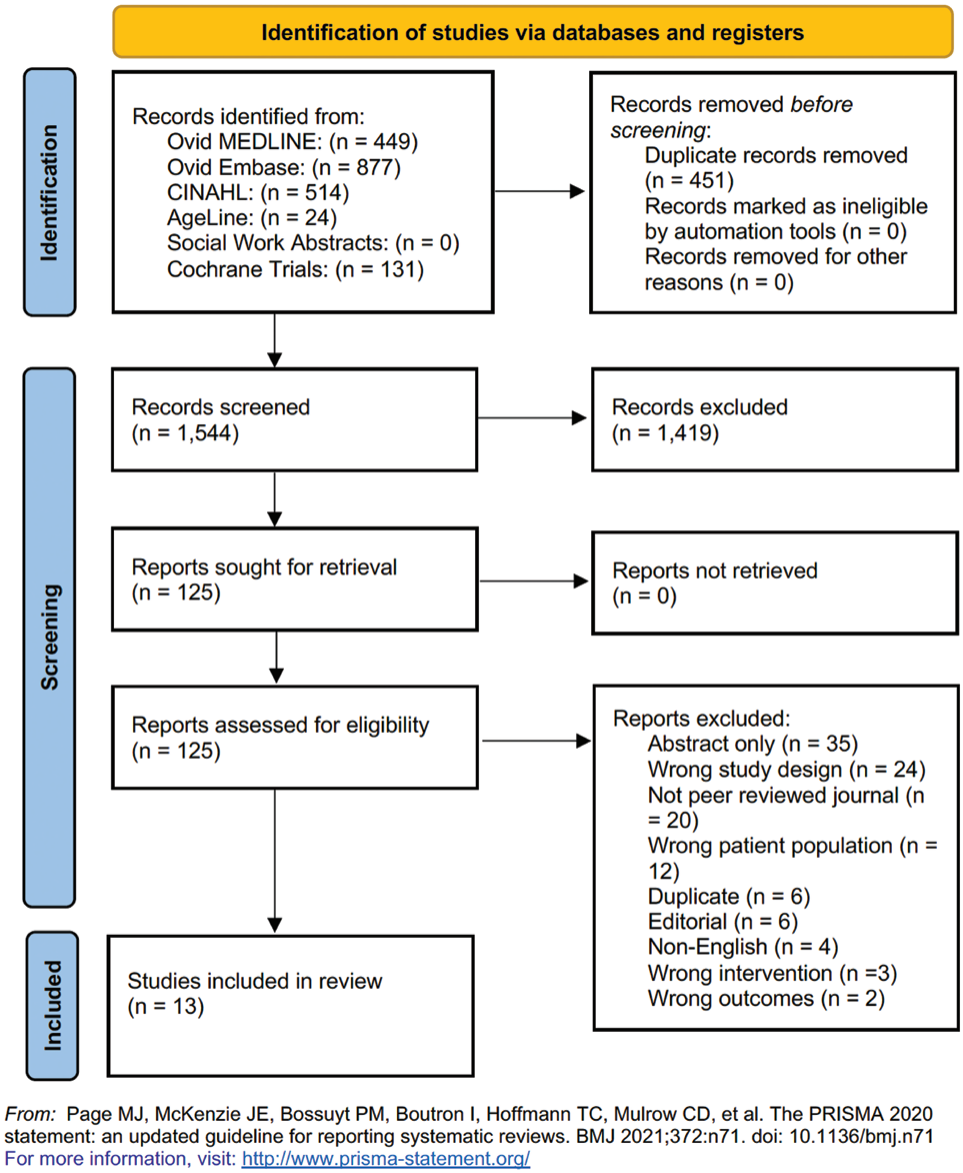
Preferred Reporting Items for Systematic Reviews and Meta-Analyses 2020 flow diagram for new systematic reviews which included searches of databases and registers only.

The PRISMA flow diagram is shown in Figure 2. We retrieved 2,160 studies from scholarly databases, and 487 were automatically excluded as duplicate records. During the title and abstract screening, 1,525 studies were excluded. During the full-text screening of the remaining 148 studies, 133 studies were excluded. After applying eligibility criteria, 15 articles were included in the review.

### Participants and Settings

Several studies were published by the same author group, reporting on feasibility study results, and primary and secondary findings from a larger trial. Specifically, 9 of the 15 studies were published by just four research groups. Most (8 of 15) studies included at least one urban site. Five of the studies also took place in suburban settings and three in rural settings. More than half (9 of 15) of the studies were based in the United States, with the remainder taking place in Canada, Germany, or the United Kingdom.

Five articles reported on randomized controlled trials (RCTs); three were cluster randomized and two randomized at the patient level. Seven studies employed observational study designs, including retrospective and prospective case series and cohort studies, and two employed interventional, nonrandomized designs. Eight studies involved a two-arm comparison.

Sample sizes ranged from 32 to 6,265 participants. The mean or median age of study participants ranged from 67 to 88, and gender breakdown ranged from 36% to 85% female. Most studies did not describe participants’ race, ethnicity, or socioeconomic status. The three studies reporting race/ethnicity data included predominantly White non-Hispanic participants (Feldman et al., 2021; Jacobsohn et al., 2022; Shah et al., 2018). Three described the participants as low-income (Agarwal et al., 2017, 2018, 2019).

Many (9 of 15) studies targeted patients who frequently utilized the health system (“high utilizers”) and/or patients with a recent inpatient admission, low-income patients, and patients with multiple comorbidities. Two interventions focused on one acute condition, such as any individual with signs and symptoms of acute coronary syndrome (Brokmann et al., 2016; Felzen et al., 2019). Six interventions specifically targeted adults over age 65, but the average age of participants in all studies was 65 or older due to the prevalence of these inclusion criteria in older adult populations.

### MIH Interventions

Most studies (12 of 15) included an in-person visit to patient homes; however, one intervention (reported in three studies) was based out of mobile clinics set up in low-income housing units specifically for older adults (Agarwal et al., 2017, 2018, 2019). Some (5 of 15) were delivered as part of a comprehensive care coordination effort, but most (10 of 15) were described as stand-alone interventions. Most (13 of 15) of the programs initiated an MIH visit through a 9-1-1 or emergency response call center, although two were prescheduled home visits (Feldman et al., 2021; Roeper et al., 2018).

Training for paramedics included in-person and online didactic education, clinical shadowing, and skills-based training and evaluation. The amount of training between programs varied from brief education sessions to multiple days; one study asked CPs to enroll in a semester-long course (Myers et al., 2020). CP evaluations and treatment activities also varied and included obtaining vital signs and electrocardiograms, risk assessments and home safety assessments, blood work and point-of-care testing, medication reconciliation and education, and history and physical exams. Interventions included administering medications (including intravenous medications) and supplemental oxygen, providing health education and coaching, coordinating referrals or communication with additional services and the care teams, and wound care. Half (7 of 15) incorporated telemedicine with a supervising physician during the visit, including video conferencing (Abrashkin et al., 2019, 2021; Brokmann et al., 2016; Feldman et al., 2021; Felzen et al., 2019; Kant et al., 2018; Myers et al., 2020). Eight studies reported a structured follow-up plan after the MIH visit (Abrashkin et al., 2019, 2021; Feldman et al., 2021; Jacobsohn et al., 2022; Myers et al., 2020; Quatman-Yates et al., 2021; Shah et al., 2018).

### Synthesis of Study Findings

#### EMS call volume

Five studies reported significant reductions in urgent EMS call volume within treatment arms receiving an MIH intervention. Agarwal et al. (2017) reported a 25% decreased in call volume, Agarwal et al. (2018) reported a mean monthly difference of −0.88 calls (95% confidence interval [CI] −0.45 to −1.30) per 100 apartment units/month in the MIH arm versus control, and Snooks et al. (2017) reported a small but significant mean adjusted difference in calls (−0.0045; 95% CI: −0.0073 to −0.0017%) in the MIH arm versus control. Agarwal et al. (2019) reported no difference in calls in the intention-to-treat analysis but reported a decline in calls when removing two sites with eligibility changes during the study (–0.90 calls [95% CI: –1.54 to –0.26] per 100 apartment units/month). Quatman-Yates et al. (2021) only examined fall-related calls, but also reported a decrease (relative risk: 0.63 [95% CI: 0.40 to 0.75]).

#### Immediate ED transports

Three studies examined immediate patient transports to the ED. They reported significant reductions overall (Kant et al., 2018), in high acuity responses (Abrashkin et al., 2019), and in fall-related transports during an initial evaluation (Quatman-Yates et al., 2021). Studies reported ED transport rates between 6% and 18% (Abrashkin et al., 2019, 2021; Kant et al., 2018), and one study reported a 51% reduction in fall-related ED transports during the initial evaluation (Quatman-Yates et al., 2021).

#### Subsequent ED visits

Five studies examined subsequent ED visits after completion of the MIH intervention; three examined within- or between-group differences. One study showed 15% of participants visited the ED within 2 weeks of an initial MIH evaluation (Kant et al., 2018). Another study found 33% of patients went to the ED within 30 days of initial hospital discharge (Feldman et al., 2021). Two studies reported significantly fewer within-group ED visits 6 months post-MIH (Myers et al., 2020; Roeper et al., 2018). One study reported no difference in 30-day ED revisits between the control and treatment groups (Jacobsohn et al., 2022).

#### Subsequent hospitalizations

Six studies examined subsequent hospitalizations, with mixed results. One study reported significant within-group reductions in hospitalizations among patients receiving MIH, but used a control arm with a lower baseline hospitalization rate, making between-group comparisons challenging (Roeper et al., 2018). Another reported a 15% all-cause readmission rate among its stage C heart failure within 30 days of discharge (Feldman et al., 2021). Two other studies reported no difference in hospitalization rates (Myers et al., 2020; Snooks et al., 2017). Hospitalization rates ranged from 14% to 40% depending on disease severity (e.g., heart failure stage), time frame (2 weeks vs 30 days), and type of readmission (disease-specific vs all-cause; Feldman et al., 2021; Kant et al., 2018).

#### CP care quality and processes

Five studies examined adverse event rates and safety, including fidelity to treatment algorithms and protocols. One study reported either no difference or greater adherence to established protocols in novel MIH interventions compared to non-CPs (Brokmann et al., 2016). Two studies reported low rates of adverse events (0%–1%; Feldman et al., 2021; Felzen et al., 2019). Completion rates of planned outreach by CPs were between 84% and 93% (Shah et al., 2018), and 75% completion rate of documentation in medical records within 2 weeks (Kant et al., 2018).

#### Patient and provider satisfaction

Five studies examined satisfaction among clinicians (two studies), patients (three studies), and caregivers (two studies); all reported high satisfaction among all groups (Abrashkin et al., 2019, 2021; Myers et al., 2020; Shah et al., 2018; Snooks et al., 2017). Patients particularly valued interpersonal aspects of MIH care (Snooks et al., 2017) and reported a strong desire to use MIH in the future (Abrashkin et al., 2019). In one study, clinicians reported that video-enabled telemedicine enhanced their clinical evaluation (Abrashkin et al., 2021).

#### Additional patient outcomes

Eight studies examined a variety of other patient outcomes after MIH interventions. Three studies reported significant improvements in health-related quality of life and quality-adjusted life years (Agarwal et al., 2018, 2019), systolic and diastolic blood pressure (Agarwal et al., 2017, 2018, 2019), and diabetes risk scores (Agarwal et al., 2018). Two studies reported significantly higher rates of follow-up with outpatient clinicians (Jacobsohn et al., 2022) or relevant services (e.g., fall service; Snooks et al., 2017), and one reported that 88% of patients attended an outpatient clinic visit within 18 days of the MIH visit (Kant et al., 2018). Patient activation in one study (Roeper et al., 2018) and recall of concerning signs and symptoms in one study (Jacobsohn et al., 2022) were significantly higher among patients receiving MIH, but there was no difference in medication adherence in one study (Jacobsohn et al., 2022).

#### Cost

Three studies examined cost savings. One study estimated the mean savings of MIH to be over $32,000, which the authors related to reduced EMS call volume (Agarwal et al., 2017). Two studies compared the mean cost of MIH to usual EMS care; one UK-based study reported no significant difference (Snooks et al., 2017) while another U.S.-based study estimated a net savings of over $2,400,000 over 6 months (Roeper et al., 2018).

#### Telemedicine outcomes

Of the seven studies reporting on MIH interventions that included a telemedicine component, only two studies reported outcomes directly related to telemedicine. One study reported few technology malfunctions (0.3%–2%; Felzen et al., 2019). Another reported that ED transport rates were not affected by physician use of video-enabled telemedicine (Abrashkin et al., 2021).

## Discussion

As MIH programs become more common both in the United States and internationally, many programs have significant potential to facilitate successful transitions of care and improve the timeliness and quality of posthospital care for community-dwelling older adults. In this systematic review of MIH in patients over 65 years of age, multiple studies demonstrated that MIH led to a reduction in EMS call volume (Agarwal et al., 2017, 2018, 2019; Quatman-Yates et al., 2021; Snooks et al., 2017) and ED transport rates (Abrashkin et al., 2019; Kant et al., 2018; Quatman-Yates et al., 2021). Reductions in call volumes and transports have been major goals for many programs, independent of age, and have proven to be successful in the low-acuity patients (Somers et al., 2020; Tyano et al., 2021). These results are therefore important to health care systems seeking to reduce ED overcrowding and hospital bed utilization, as well as older adults who can avoid the “toxicities of hospitalization,” including in-hospital mortality, by receiving care in the home versus a hospital setting (Herbert et al., 2017; LaMantia et al., 2010; Launay et al., 2014; Strum et al., 2021).

MIH can reduce fall-related calls to EMS, which is a common reason for 9-1-1 activations among older adults (Quatman-Yates et al., 2021). Similarly, our review showed that MIH programs can be successfully employed in complex medical patients. For instance, Feldman et al. (2021) found the incidence of 30-day all-cause readmissions was less than half in the stage D heart failure group and 15% in the stage C heart failure group. They also found no adverse events in either group and no deaths reported, which illustrates the safety of programs in high-risk patient populations. The safety of these programs was also demonstrated by Kant et al. (2018) who showed no hospitalizations of the >90-year-old patients on either the day of their MIH visit or at 2 weeks following the initial visit. Finally, one of the most important benefits of the studies was satisfaction with MIH care reported by both the patients and the providers, as satisfaction supports the sustainability of the programs. For example, Abrashkin et al. (2019) reported that 100% of patients and caregivers agreed or strongly agreed to using MIH in a future medical emergency.

While MIH has shown promise improving multiple outcomes for older adults, it is challenging to draw definitive conclusions because of the heterogeneity in MIH interventions, including the scope of evaluation and treatment, CP training, physician oversight, post-visit follow-up, and use of telemedicine. Variability between programs may in part be due to the laws regulating CP training and licensure vary by state and country such that CP scope of practice, standards for credentialing, and whether MIH visits should be reactive or preventative vary widely (Glenn et al., 2018; Hollander & Sharma, 2021; National Association of Emergency Medical Technicians, 2018). Flexible definitions of MIH allow program leadership to tailor interventions to the unique needs and constraints of their respective community, but a lack of standardized best practices and metrics for measuring success could be a reason for the mixed effects on major endpoints such as health care utilization and health outcomes observed.

Therefore, this systematic review highlights the importance of implementing guidelines regarding CP training, program coordination, and quality-based metrics used to measure effectiveness. It will also be important for studies to thoroughly describe MIH interventions using accepted implementation frameworks such as Reach Effectiveness Adoption Implementation Maintenance (Glasgow et al., 1999) and Consolidated Framework for Implementation Research (Damschroder et al., 2009); these frameworks help identify metrics that can be used to improve success and outcomes of a respective health care program (Holtrop et al., 2018; King et al., 2020). Moreover, high-quality evidence from well-powered, RCTs is needed to quantify the effectiveness of MIH (Kim & Basu, 2021). Few of the studies we reviewed employed RCT designs. Multiple RCTs are currently underway that could provide critical evidence on the effectiveness of MIH in older adult populations (Masterson Creber et al., 2022).

The feasibility and effectiveness of incorporating telemedicine within MIH interventions remain an understudied area. Half (seven) of the included articles reported on MIH interventions that used telemedicine; five described video conferencing, one described telephonic communication, and one did not specify the telemedicine modality. However, only two of these studies reported outcomes directly related to the telemedicine component. Felzen et al. (2019) examined technical performance of the telemedicine platform and reported overall low rates of voice communication or data transmission malfunctions or complete system failures. Abrashkin et al. (2021) examined physician satisfaction with telemedicine, finding that video-enabled telemedicine visits enhanced physicians’ clinical evaluation 85% of the time, but were not more associated with odds of ED transport compared to telephonic visits. While these studies suggest positive effects of inclusion of video-enabled telemedicine into MIH interventions, there were too few to draw meaningful conclusions in this review.

A potential benefit of incorporating telemedicine into MIH is the potential to ameliorate known disparities in telemedicine access among older adults who identify as racial or ethnic minorities, live in rural areas, or report low socioeconomic status (Chunara et al., 2021; Goldberg et al., 2022; Lame et al., 2021; Litchfield et al., 2021; Mitchell et al., 2019; Weber et al., 2020). Individuals delivering MIH may address these issues by providing facilitated telemedicine for community-dwelling older adults by supplying reliable Internet access and technology along with real-time technical support (Elbaz et al., 2021; Goldberg et al., 2022; Lam et al., 2020).

It is possible that the use of telemedicine in MIH has risen since the coronavirus disease 2019 (COVID-19) pandemic. Despite conducting our search in 2021, the most recent studies ended prior to the pandemic beginning. Some have suggested MIH filled a critical gap in health care services throughout the COVID-19 pandemic by providing an alternative to hospital- and clinic-based care, especially amidst strict public health measures (including social distancing and shelter-in-place ordinances) and corresponding gaps in health care outcomes (Gaudino et al., 2020; Patt et al., 2020; Weigel et al., 2020). MIH was recognized as a way to simultaneously decompress overburdened emergency rooms and inpatient units, while still providing the care that high-risk older adults need (Constantine et al., 2021). This is particularly important because of the evidence of excess non-COVID related deaths during the pandemic attributed to decreased hospital capacity, delayed surgical interventions, and patient fear of contracting COVID-19, especially among older adults (Mafham et al., 2020; Mesnier et al., 2020; Naccarato et al., 2020; Rudilosso et al., 2020). When published, studies of MIH interventions during the pandemic, particularly those incorporating telemedicine, will be informative in future pandemic preparedness efforts.

A major limitation of this review is the wide variability in MIH intervention implementations, study designs, and outcomes assessed in the included studies, which is evident in the range of Downs and Black quality scores. This variability makes synthesizing the evidence and drawing generalizable conclusions about the state of the science challenging. Furthermore, both the absence of consistent outcome measures and the wide variability of study designs for MIH made it difficult to perform standardized data abstraction or synthesis. MIH is an evolving area with new performance metrics and methodology, making it challenging to fully capture all pertinent studies in this area despite employing a systematic review process. Finally, many of the studies included in this review lack sufficient data on the socioeconomic, racial, and ethnic backgrounds of participants, which limits our understanding of the impact MIH may have on various sociodemographic subgroups. Improved reporting and analysis of such subgroups is of high importance, as MIH has the potential to reach sociodemographic subpopulations less likely to access nontraditional forms of care such as telemedicine (Faverio, 2022).

## Conclusion

MIH interventions represent innovative care-delivery models that may allow older adults to remain at home and avoid the “toxicities” associated with multiple hospitalizations. This review evaluating MIH interventions among adults aged 65 and older demonstrated clear reductions in EMS call volume and ED transport rates, and possible improvements in patient and provider satisfaction and costs. However, we also found wide variability in the characteristics of the MIH interventions evaluated, which reflects the natural adaptations of MIH interventions to a range of unique patient populations and environments internationally over time. As such, quality improvement models supported by high-quality, rigorous research are needed to fully recognize the potential for MIH to improve health outcomes and reduce unnecessary health care utilization among older adults.

## Supplementary Material

igad017_suppl_Supplementary_Material

## Transparency and Openness Promotion

For replication purposes, the original studies included in this review have been accurately cited and the tables describing the synthesis of results have been published with this manuscript as supplementary material. A PRISMA flow diagram and checklist have also been included. Other materials used during this review not included in the publication, such as the data extraction form, are available upon request to the corresponding author. This study has been preregistered on an independent, institutional registry, Open Science Forum: https://osf.io/ufn47/.
