# Correction to: Mobile Integrated Health Interventions for Older Adults: A Systematic Review

**DOI:** 10.1093/geroni/igad053

**Published:** 2023-06-22

This is a correction to: Nathan Louras, MD, Meghan Reading Turchioe, PhD, MPH, RN, Leah Shafran Topaz, MSc, BPT, Michelle R. Demetres, MLIS, Melani Ellison, BS, Jamie Abudu-Solo, BS, Erik Blutinger, MD, MSc, Kevin G Munjal, MD, MPH, Brock Daniels, MD, MPH, Ruth M Masterson Creber, PhD, MSc, RN, Mobile Integrated Health Interventions for Older Adults: A Systematic Review, *Innovation in Aging*, Volume 7, Issue 3, 2023, igad017, https://doi.org/10.1093/geroni/igad017

In the article, “Mobile Integrated Health Interventions for Older Adults: A Systematic Review,” the following author was omitted from the author list in error: Michelle R. Demetres, MLIS. This error has been corrected.

