# Supplementary material for: Mobile Integrated Health Interventions for Older Adults: A Systematic Review
Source: Innov Aging. 2023 Mar 1;7(3):igad017. doi: 10.1093/geroni/igad017 (PMC10114527; doi:10.1093/geroni/igad017)
Supplement: igad017_suppl_Supplementary_Material [file igad017_suppl_supplementary_material.docx]

**Online Supplementary Material**

Detailed search strategy, study characteristics, and quality appraisal results

**Ovid MEDLINE:**

Ovid MEDLINE (ALL – 1946 to present)

Searched on December 7, 2021

No language, publication date, or article type restrictions

| **Line #** | **Search** |
| --- | --- |
| 1 | Community Health Services/ or Mobile Health Units/ or Telemedicine/ |
| 2 | Emergency Medical Technicians/ or Ambulances/ |
| 3 | 1 and 2 |
| 4 | (communit* paramedic* or communit* based paramedic* or paramedic* practitioner* or telehealth-enabled emergency medic* or mobile integrated healthcare or mobile integrated health care or MIH-CP or CP-MIH or prehospital case management or pre-hospital case management or EMS-based case management or EMS-based outreach).tw. |
| 5 | 3 or 4 |

**Ovid EMBASE:**

Embase (1974 to 2020 November 30)

Searched on December 7, 2021

No language, publication date, or article type restrictions

| **Line #** | **Search** |
| --- | --- |
| 1 | community care/ or telemedicine/ |
| 2 | paramedical personnel/ or ambulance/ or ambulance transportation/ |
| 3 | 1 and 2 |
| 4 | (communit* paramedic* or communit* based paramedic* or paramedic* practitioner* or telehealth-enabled emergency medic* or mobile integrated healthcare or mobile integrated health care or MIH-CP or CP-MIH or prehospital case management or pre-hospital case management or EMS-based case management or EMS-based outreach).tw. |
| 5 | 3 or 4 |

**Cochrane Library:**

Date Run: 07/12/2021

ID Search

#1 (communit* paramedic* or communit* based paramedic* or paramedic* practitioner* or telehealth-enabled emergency medic* or "mobile integrated healthcare" or "mobile integrated health care" or "MIH-CP" or "CP-MIH" or "prehospital case management" or "pre-hospital case management" or "EMS-based case management" or "EMS-based outreach"):ti,ab

**CINAHL (EBSCO)**

Searched on December 7, 2021

| **#** | **Query** |
| --- | --- |
| S7 | S5 OR S6 |
| S6 | TI ( "community paramedicine" OR "community paramedic" OR "community based paramedicine" OR "paramedicine practitioner" OR "telehealth-enabled emergency medicine" OR "mobile integrated healthcare" OR "mobile integrated health care" OR "MIH-CP" OR "CP-MIH" OR "prehospital case management" OR "pre-hospital case management" OR "EMS-based case management" OR "EMS-based outreach" ) OR AB ( "community paramedicine" OR "community paramedic" OR "community based paramedicine" OR "paramedicine practitioner" OR "telehealth-enabled emergency medicine" OR "mobile integrated healthcare" OR "mobile integrated health care" OR "MIH-CP" OR "CP-MIH" OR "prehospital case management" OR "pre-hospital case management" OR "EMS-based case management" OR "EMS-based outreach" ) |
| S5 | S3 AND S4 |
| S4 | (MH "Emergency Medical Technicians") OR (MH "Ambulances") |
| S3 | S1 OR S2 |
| S2 | (MH "Telemedicine") OR (MH "Telehealth") |
| S1 | (MH "Community Health Services") |

**AGELINE (EBSCO)**

Searched on December 7, 2021

| **#** | **Query** |
| --- | --- |
| S3 | S1 OR S2 |
| S2 | (DE "Public Health Services") AND (DE "Emergency Health Services") |
| S1 | TI ( "community paramedicine" OR "community paramedic" OR "community based paramedicine" OR "paramedicine practitioner" OR "telehealth-enabled emergency medicine" OR "mobile integrated healthcare" OR "mobile integrated health care" OR "MIH-CP" OR "CP-MIH" OR "prehospital case management" OR "pre-hospital case management" OR "EMS-based case management" OR "EMS-based outreach" ) AND AB ( "community paramedicine" OR "community paramedic" OR "community based paramedicine" OR "paramedicine practitioner" OR "telehealth-enabled emergency medicine" OR "mobile integrated healthcare" OR "mobile integrated health care" OR "MIH-CP" OR "CP-MIH" OR "prehospital case management" OR "pre-hospital case management" OR "EMS-based case management" OR "EMS-based outreach" ) |

**Social Work Abstracts (Ovid)**

Searched on December 7, 2021

1 (communit* paramedic* or communit* based paramedic* or paramedic* practitioner* or telehealth-enabled emergency medic* or mobile integrated healthcare or mobile integrated health care or MIH-CP or CP-MIH or prehospital case management or pre-hospital case management or EMS-based case management or EMS-based outreach).tw.

| **Supplemental Table 1.** Additional study details | | | | | | | |
| --- | --- | --- | --- | --- | --- | --- | --- |
| **Study** | **Age** | **Gender** | **Race/ ethnicity** | **Sample size** | **Visit sample size** | **Secondary outcome measures** | **Secondary results** |
| Abrashkin et al. (2019) | Mean 86 years | 65% female | Not reported | 1,159 | 2,378 | - ED presentations 48 hours post-visit - Patient or caregiver satisfaction | - 5.7% of high acuity responses not transported to the ED by CP’s presented to the ED within 48 hours post-visit. - Patient/ caregiver satisfaction was high, and 100% agreed or strongly agreed they would use the CP service in a future medical emergency. |
| Abrashkin et al. (2021) | Median 88 years | 63% female | Not reported | 1,068 | 1,927 | - Perceived enhancement in evaluation provided by the video communication - Odds of ED transport associated with perceived enhancement | - Physicians endorsed that video communication enhanced their clinical evaluation 85% of the time. - There was no association between physicians’ report that video enhanced their clinical evaluation and ED transport (OR 1.06; 95% CI 0.62 to 1.80). |
| Agarwal et al. (2017) | Mean 72 years | 68% female | Not reported | 79 | 1,365 | - Post-hoc cost analysis | - The reduced EMS call volume was associated with an estimated average savings of $32,520 (range: $9,980-$45,080). |
| Agarwal et al. (2018) | Intervention: mean 74 years, Control: mean 71 years | Intervention: 76% female, Control: 74% female | Not reported | 1,092 | Not reported | - QALYs - HRQOL - Lifestyle risk-factor measures including blood pressure | - Significant increase in QALYs in intervention vs. control (mean difference 0.09, 95% CI: 0.01-0.17) - Significant increase in HRQOL domain “ability to perform usual activities” in intervention vs. control (OR 2.6, 95% CI 1.2 to 5.8) - Significant decrease in systolic (mean change 5.0 mmHg, 95% CI 1.0 to 9.0) and diastolic (mean change 4.8 mmHg, 95% CI 1.9 to 7.6) blood pressure in intervention vs. control |
| Agarwal et al. (2019) | Intervention: mean 74 years, Control: mean 70 years | Intervention: 80% female, Control: 72% female | Not reported | 4,081 | Not reported | - QALYs - HRQOL - Lifestyle risk-factor measures including blood pressure | - Significant increase in QALYs in intervention vs. control (mean difference 0.06, 95%CI: 0.02 to 0.10) - Significant increase in three HRQOL domains (self-care, usual activities, pain, and discomfort) in intervention vs. control - Significant decrease in systolic (mean change 3.65 mmHg, 95% CI: 2.37 to 4.94) and diastolic (mean change 2.03 mmHg, 95% CI: 1.00 to 3.06) blood pressure in intervention vs. control |
| Brokmann et al. (2016) | Mean 68 years (both groups) | 36% female (both groups) | Not reported | 39 | 39 | - None | N/A |
| Feldman et al. (2021) | Stage C HF: mean 70 years, Stage D HF: mean 67 years | 25% female (both stage C and D HF) | Stage C HF: 90% White, 10% Other; Stage D HF: 85% White, 10% Black, 5% Other | 40 | 82 | - 30-day HF readmissions - ED visits - Unplanned office visits - Adverse events | - The incidence of 30-day HF readmissions was 5% for heart failure stage C patients, and 20% for heart failure stage D patients - The incidence of patient-initiated ED visits was 15% for heart failure stage C patients, and 45% for heart failure stage D patients - The incidence of unplanned office visits was 20% for both patient cohorts - There were no adverse events |
| Felzen et al. (2019) | Median 70 years | 53% female | Not reported | 6,265 | 6,265 | None | N/A |
| Jacobsohn et al. (2021) | Mean 72 years | 53% female | 7% non-white, 2% Hispanic | 1,756 | 726 | - Outpatient follow-up - Knowledge of “red flag” signs and symptoms - Medication adherence | - Significantly increased odds of in-person follow-up with outpatient clinicians during the week following discharge (adjusted OR = 1.24, 95% CI: 1.01 to 1.51) in intervention vs. control. - Significantly increased odds of recalling at least one red flag from ED discharge instructions (adjusted OR = 1.34, 95% CI: 1.05 to 1.71) in intervention vs. control. - No significant difference in medication adherence. |
| Kant et al. (2018) | Mean 88 years | 85% female | Not reported | 35 | 40 | - Documentation practices - Post-visit clinic follow-up | - 30 visits (75%) had notes scanned into the patients’ medical records within two weeks of CP visit, and 3 (8%) were scanned in more than two weeks later - 7 visits (18%) never had notes scanned into the medical record - 35 visits (88%) were followed by a clinic visit within a mean 18 (SD 19) days |
| Myers et al. (2020) | Median age 76 years | 69% female | Not reported | 32 | 412 | - Provider satisfaction | - Providers endorsed high satisfaction with the service on a multi-item satisfaction survey. |
| Quatman-Yates (2021) | Median 77 years | 59% female | Not reported | Not reported | 892 | None | N/A |
| Roeper et al. (2018) | Intervention: mean 74 years, Control: mean 75 years | Intervention: 58% female, Control: 57% female | Not reported | 2,315 | Not reported | - ED visits - Inpatient hospitalizations - Patient activation | - Over six months, there were significantly fewer ED visits in the intervention vs. control arm (–9.28 vs. 4.62 visits per 1,000, p=0.003) - Over six months, there were significantly fewer hospitalizations in the intervention vs. control arm (–5.91 vs. 5.77 visits per 1,000, p<0.0001) - Statistically significant increase in patient activation within the intervention arm (7.5% increase; p<0.01) |
| Shah et al. (2018) | Median 71 years | 57% female | 91% White | 853 | 354 | None | N/A |
| Snooks et al. (2017) | Mean 82 years | 63% female | Not reported | 4,655 | 4,655 | - Referrals, documentation completion rates, visit length at time of CP visit - Subsequent falls, hospitalization, patient-reported outcomes, patient satisfaction, and care costs after CP visit | - Significantly more referrals to fall service in intervention vs. control (8% vs. 1%). - Significantly higher patient satisfaction with interpersonal aspects of care in intervention vs. control. - No difference in mean intervention cost. |
| *RCT: CP: community paramedic; ED: Emergency department; HF: Heart failure; HRQOL: Health-related quality of life; QALY: Quality-adjusted life year.* | | | | | | | |

| **Supplemental Table 2.** Description of MIH programs in included studies | | | | | | | |
| --- | --- | --- | --- | --- | --- | --- | --- |
| **Study** | **Program description** | **CP Training** | **Visit components** | | | **Clinical oversight** | **Post-visit follow-up** |
|  |  |  | **Evaluation** | **Treatment** | **Telemedicine** |  |  |
| Abrashkin et al. 2019; 2021 | CP intervention dispatched from nurse-run call center as part of a broader advanced illness management program from homebound individuals | 40 hours of didactic and observation-based instruction in geriatrics and home-based primary care | - Physical exam - Vital signs (12-lead ECGs, vital signs) - Blood glucose monitoring | - Medications: IV, IM, oral, nebulizers | Video or telephone conferencing as needed | Clinic physicians, including internists, family medicine practitioners, geriatricians, and palliative care–trained physicians credentialed to provide online medical control | Telephone call by registered nurse within 6 hours of visit |
| Agarwal et al. 2017; 2018; 2019 | Weekly drop-in clinic based in subsidized older adult apartment buildings | Online interactive modules on chronic diseases,  their risk factors, risk assessment using validated  tools, and health promotion methods (4 hours); webinars for database training (1 hour); in-person observation (2–3 hours) | - Blood pressure - Diabetes risk assessment - Fall risk assessment | - Provide health education health education and referrals to community resources - Communicate health information and risks to family physicians - Immediate referral to family physician or ED in emergency situations | Not described | Not described | Not described |
| Brokmann et al. (2016); Felzen et al. (2019) | CP intervention to treat acute coronary syndromes in the field prior to ED transport | Standardized eight-hour training program to learn the use of the technical system, the medical concept  of teleconsultation including indications for teleconsultation  and communication skills | - Vital signs (12-lead ECGs, vital signs) | - IV medications (aspirin, heparin, morphine) - Supplemental oxygen | Real-time transmission of ECGs and vital signs, and video conference | “Tele-EMS” physicians with critical care and EMS experience provided synchronous guidance to CPs | Not described |
| Feldman et al. (2021) | Post-hospital discharge CP intervention including both pre-scheduled (2- and 15-days post-discharge) and urgent visits dispatched from a call center. | Two-day training sessions covering CP roles, social needs assessment, clinical assessment and treatment, communication and documentation, and “mock drills” simulating clinical scenarios. Also completed human subjects research training and shadowed a heart failure nurse practitioner. | - Social needs assessment - Home safety assessment - Physical assessment - Vital signs | - IV diuretics - Topical nitroglycerin | Video conferencing with physician or heart failure specialist | Physician, heart failure specialist, or nurse practitioner ordered medications and requested transport to ED as needed | Additional house calls and other home-based interventions if requested by heart failure specialist |
| Kant et al. (2018) | Nurse practitioners or physician assistants plus CPs provide episodic care with ED physician consultations as needed to patients seen at an academic geriatric clinic. | Not described | - Bloodwork - Basic imaging | - Medications (including parenteral), - IV fluids - Minor procedures such as laceration repair and Foley management | On-call ED physician available as needed (platform not described) | Not described | Not described |
| Myers et al. (2020) | Home-based CP intervention based on primary care physician referrals for high utilizer patients | Distance education CP course consisting of classroom time (72 hours), online content (72 hours), and clinical time (196 hours) lasting one semester. | - Physical exam - Medication reconciliation - Home assessment - Point of care testing - Bloodwork | - Wound care | Phone call to ordering or on-call physician | Physician chart review after visit completion. | Additional CP visits if requested by primary care physician |
| Roeper et al. (2018) | Home-based pre-scheduled and urgent CP visits delivered as part of broader care coordination program | Not described | - History - Medication reconciliation | - Outpatient appointment follow-up - Self-management coaching - Medication instruction - Diagnosis-related education - Additional physician-directed EMS care (not described in detail) | Not described | Physician directed all EMS care | Care coordinators continued patient monitoring |
| Quatman-Yates (2021) | Home-based CP intervention to support fall prevention among high-risk older adults | Training provided; detail not described. | - History - Medication reconciliation - Vital signs - ECG - Fall risk, cognitive, and mental health assessments | - Deliver immediate fall-risk reduction interventions and document planned follow-up strategy | Not described | Not described | Algorithm-directed routine monitoring including phone calls and home visits |
| Shah et al. (2018), Jacobson et al. (2021) | 4-week hospital-to-home program using CP’s to promote self-management skills among recently hospitalized older adults | Orientation to program details and protocols (2 days), motivational interview coaching (10-15 hours), geriatrics-specific education (10 hours), shadowing ED discharge process (8 hours), general education about community paramedicine (2 hours) | - Medication reconciliation - History | - Coaching related to self-management and awareness of “red flag” signs and symptoms | Not described | Not described | Up to three telephone calls reinforcing self-management goals and patient education |
| Snooks et al. (2017) | Home-based CP intervention to assess older adults who have fallen and determine an appropriate course of action (ED vs. community-based fall service) | One centralized meeting to discuss protocol and provide training materials; CP training provided locally within each EMS station | - Physical assessment - History - POC testing - Vital signs - ECG | - Referral to home-based fall service as appropriate | Not described | Not described | Not described |
| *Studies reporting on the same MIH intervention are group together.  *CP: community paramedic; ECG: Electrocardiogram; ED: Emergency department; EMS: Emergency medical services; IM: Intramuscular; IV: intravenous; MIH: Mobile integrated health; POC: point-of-care.* | | | | | | | |

| **Supplemental Table 3.** Quality appraisal results | | | | | | | | | | | | | | | | | | | | | | | | | | | | |  |
| --- | --- | --- | --- | --- | --- | --- | --- | --- | --- | --- | --- | --- | --- | --- | --- | --- | --- | --- | --- | --- | --- | --- | --- | --- | --- | --- | --- | --- | --- |
| **Study** | **Q1** | **Q2** | **Q3** | **Q4** | **Q5** | **Q6** | **Q7** | **Q8** | **Q9** | **Q10** | **Q11** | **Q12** | **Q13** | **Q14** | **Q15** | **Q16** | **Q17** | **Q18** | **Q19** | **Q20** | **Q21** | **Q22** | **Q23** | **Q24** | **Q25** | **Q26** | **Q27** | **Score** | |
| Abrashkin et al. (2019) | 1 | 1 | 1 | 1 | 0 | 1 | 0 | 1 | 0 | 0 | 1 | 1 | 1 | 0 | 0 | 0 | 0 | 1 | 1 | 1 | 0 | 0 | 0 | 0 | 0 | 0 | 0 | 12 |  |
| Abrashkin et al. (2021) | 1 | 1 | 1 | 1 | 1 | 1 | 1 | 0 | 0 | 1 | 1 | 1 | 1 | 0 | 0 | 1 | 0 | 1 | 1 | 1 | 1 | 1 | 0 | 0 | 1 | 0 | 0 | 18 |  |
| Agarwal et al. (2017) | 1 | 1 | 1 | 1 | 0 | 1 | 1 | 1 | 0 | 1 | 1 | 0 | 1 | 0 | 0 | 1 | 1 | 1 | 1 | 1 | 1 | 1 | 0 | 0 | 0 | 0 | 1 | 18 |  |
| Agarwal et al. (2018) | 1 | 1 | 1 | 1 | 1 | 1 | 1 | 0 | 1 | 1 | 1 | 1 | 1 | 0 | 0 | 1 | 1 | 1 | 1 | 1 | 1 | 1 | 1 | 0 | 1 | 1 | 1 | 23 |  |
| Agarwal et al. (2019) | 1 | 1 | 1 | 1 | 1 | 1 | 1 | 1 | 1 | 1 | 1 | 1 | 1 | 0 | 0 | 1 | 1 | 1 | 1 | 1 | 1 | 1 | 1 | 0 | 1 | 1 | 1 | 24 |  |
| Brokmann et al. (2016) | 1 | 1 | 1 | 1 | 1 | 1 | 0 | 1 | 0 | 1 | 1 | 1 | 1 | 0 | 0 | 1 | 0 | 1 | 1 | 1 | 1 | 0 | 0 | 0 | 0 | 0 | 0 | 16 |  |
| Feldman et al. (2021) | 1 | 1 | 1 | 1 | 1 | 1 | 0 | 1 | 1 | 1 | 0 | 0 | 1 | 0 | 0 | 1 | 1 | 1 | 0 | 1 | 0 | 1 | 0 | 0 | 0 | 1 | 0 | 16 |  |
| Felzen et al. (2019) | 1 | 1 | 1 | 1 | 0 | 1 | 1 | 1 | 0 | 1 | 0 | 1 | 1 | 0 | 0 | 1 | 0 | 1 | 0 | 1 | 0 | 0 | 0 | 0 | 0 | 0 | 0 | 13 |  |
| Jacobsohn et al. (2021) | 1 | 1 | 1 | 1 | 1 | 1 | 1 | 1 | 1 | 0 | 1 | 1 | 1 | 0 | 1 | 1 | 1 | 1 | 1 | 1 | 1 | 1 | 1 | 0 | 1 | 1 | 1 | 24 |  |
| Kant et al. (2018) | 1 | 1 | 1 | 1 | 0 | 1 | 1 | 0 | 0 | 0 | 1 | 1 | 1 | 0 | 0 | 1 | 0 | 0 | 1 | 1 | 0 | 0 | 0 | 0 | 0 | 0 | 0 | 12 |  |
| Myers et al. (2020) | 1 | 1 | 1 | 1 | 1 | 1 | 0 | 0 | 1 | 1 | 1 | 1 | 1 | 0 | 0 | 1 | 1 | 1 | 1 | 1 | 1 | 0 | 0 | 0 | 0 | 1 | 0 | 18 |  |
| Quatman-Yates (2021) | 1 | 1 | 1 | 1 | 0 | 1 | 1 | 0 | 0 | 0 | 1 | 1 | 1 | 0 | 0 | 1 | 0 | 1 | 1 | 1 | 0 | 0 | 0 | 0 | 0 | 0 | 0 | 13 |  |
| Roeper et al. (2018) | 1 | 1 | 1 | 1 | 1 | 1 | 0 | 0 | 1 | 1 | 0 | 0 | 1 | 0 | 0 | 1 | 1 | 1 | 1 | 1 | 1 | 1 | 0 | 0 | 1 | 1 | 0 | 18 |  |
| Shah et al. (2018) | 1 | 1 | 1 | 1 | 0 | 1 | 1 | 0 | 0 | 0 | 1 | 0 | 1 | 0 | 1 | 1 | 0 | 1 | 1 | 1 | 1 | 1 | 1 | 0 | 0 | 1 | 0 | 17 |  |
| Snooks et al. (2017) | 1 | 1 | 1 | 1 | 1 | 1 | 1 | 1 | 1 | 1 | 1 | 1 | 1 | 0 | 1 | 1 | 1 | 1 | 1 | 1 | 1 | 1 | 1 | 0 | 1 | 1 | 1 | 25 |  |
| *Quality appraisal was conducted using the Downs and Black checklist.* | | | | | | | | | | | | | | | | | | | | | | | | | | | | |  |
